# SAP domain-dependent Mkl1 signaling stimulates proliferation and cell migration by induction of a distinct gene set indicative of poor prognosis in breast cancer patients

**DOI:** 10.1186/1476-4598-13-22

**Published:** 2014-02-05

**Authors:** Irem Gurbuz, Jacqueline Ferralli, Tim Roloff, Ruth Chiquet-Ehrismann, Maria B Asparuhova

**Affiliations:** 1 Friedrich Miescher Institute for Biomedical Research, Maulbeerstrasse 66, Basel CH-4058, Switzerland; 2 Faculty of Science, University of Basel, Klingelbergstrasse 50, Basel CH-4056, Switzerland

**Keywords:** Myocardin-related transcription factor-A (MRTF-A), Metastasis, Cancer progression, Prognosis, Gene regulation, Mechanical strain

## Abstract

**Background:**

The main cause of death of breast cancer patients is not the primary tumor itself but the metastatic disease. Identifying breast cancer-specific signatures for metastasis and learning more about the nature of the genes involved in the metastatic process would 1) improve our understanding of the mechanisms of cancer progression and 2) reveal new therapeutic targets. Previous studies showed that the transcriptional regulator megakaryoblastic leukemia-1 (Mkl1) induces tenascin-C expression in normal and transformed mammary epithelial cells. Tenascin-C is known to be expressed in metastatic niches, is highly induced in cancer stroma and promotes breast cancer metastasis to the lung.

**Methods:**

Using HC11 mammary epithelial cells overexpressing different Mkl1 constructs, we devised a subtractive transcript profiling screen to identify the mechanism by which Mkl1 induces a gene set co-regulated with tenascin-C. We performed computational analysis of the Mkl1 target genes and used cell biological experiments to confirm the effect of these gene products on cell behavior. To analyze whether this gene set is prognostic of accelerated cancer progression in human patients, we used the bioinformatics tool GOBO that allowed us to investigate a large breast tumor data set linked to patient data.

**Results:**

We discovered a breast cancer-specific set of genes including tenascin-C, which is regulated by Mkl1 in a SAP domain-dependent, serum response factor-independent manner and is strongly implicated in cell proliferation, cell motility and cancer. Downregulation of this set of transcripts by overexpression of Mkl1 lacking the SAP domain inhibited cell growth and cell migration. Many of these genes are direct Mkl1 targets since their promoter-reporter constructs were induced by Mkl1 in a SAP domain-dependent manner. Transcripts, most strongly reduced in the absence of the SAP domain were mechanoresponsive. Finally, expression of this gene set is associated with high-proliferative poor-outcome classes in human breast cancer and a strongly reduced survival rate for patients independent of tumor grade.

**Conclusions:**

This study highlights a crucial role for the transcriptional regulator Mkl1 and its SAP domain during breast cancer progression. We identified a novel gene set that correlates with bad prognosis and thus may help in deciding the rigor of therapy.

## Background

Most breast cancer patients die from tumor metastases and not from the primary tumor itself. Thus, the identification of genes and signaling pathways influencing the metastatic process are of utmost importance. Once the mechanisms leading to metastasis are uncovered, they can in the future serve as a rational basis for prognosis and intervention. From the beginning of its discovery, tenascin-C has been strongly associated with tumorigenesis and cancer progression in many different types of tumors (reviewed in [1,2]). Tenascin-C was not only enriched in breast cancer tissue [3,4], but its high expression was part of a gene signature of breast cancers metastasizing to the lung [5]. There is strong evidence that tenascin-C contributes to the metastatic behavior of breast cancer cells [6] by providing a niche for their settlement in the lung [7,8]. The source of tenascin-C can be the tumor cells themselves as well as the stromal cells of the cancer microenvironment. Downregulation of tenascin-C by miR-335 or shRNA in human cancer cells in a mouse xenograft model inhibits metastasis formation [7], and in tenascin-C-deficient mice, metastasis formation of tenascin-C positive cancer cells is also suppressed [9].

There are many signaling pathways inducing tenascin-C expression (reviewed in [10]). Among these, mechanical strain application *in vivo* as well as to cells in culture is a potent stimulus to induce tenascin-C expression in fibroblasts [11,12]. We have recently shown that induction of tenascin-C by cyclic mechanical strain requires the action of Mkl1 [13]. Mkl1 is a member of the myocardin-related transcription factor family (MRTF) and a well-known transcriptional co-activator of serum response factor (SRF) [14-16]. SRF target genes, which are regulated upon recruitment of MRTF cofactors, encode proteins involved in actin cytoskeletal function that can either be structural (for example, actin) or related to actin dynamics (for example, talin 1) (reviewed in [17,18]). However, Mkl1-mediated stretch-induced tenascin-C expression in fibroblasts did not require SRF, but instead depended on the potential DNA-binding SAP domain of Mkl1. This implies a novel mode of Mkl1 action as a *bona fide* transcription factor in mechanotransduction [13]. Interestingly, normal and transformed mouse mammary epithelial cells also appear to be highly sensitive to Mkl1 signaling, responding to Mkl1 overexpression with several fold induction of tenascin-C [13].

The present study was designed to find SAP-dependent Mkl1 target genes co-regulated with tenascin-C and to analyze whether such genes could be indicative of specific physiological states of cells that might be controlled by mechanotransduction. For our study, we made use of the HC11 mammary epithelial cell line. HC11 cells are capable of both self-renewal and differentiation and can be cultured for unlimited time in an undifferentiated state [19], the condition we used in our study. HC11 cells can reconstitute the ductal epithelium of a cleared mammary fat pad *in vivo* with ductal, alveolar and myoepithelial cells, illustrating their stem cell abilities [19,20]. In addition, HC11 cells contain a mutated p53 gene that not only increases the replicative potential of stem cells but confers predisposition to mammary carcinoma [21]. Undifferentiated HC11 cells share transcriptome signatures with human breast cancer [22], supporting the relevance of this model for breast cancer-related studies. We therefore concluded our study by investigating whether the genes co-regulated with tenascin-C would also be implicated in breast cancer progression.

## Results

### Screen for SAP-dependent Mkl1 target genes

**Figure 1:**
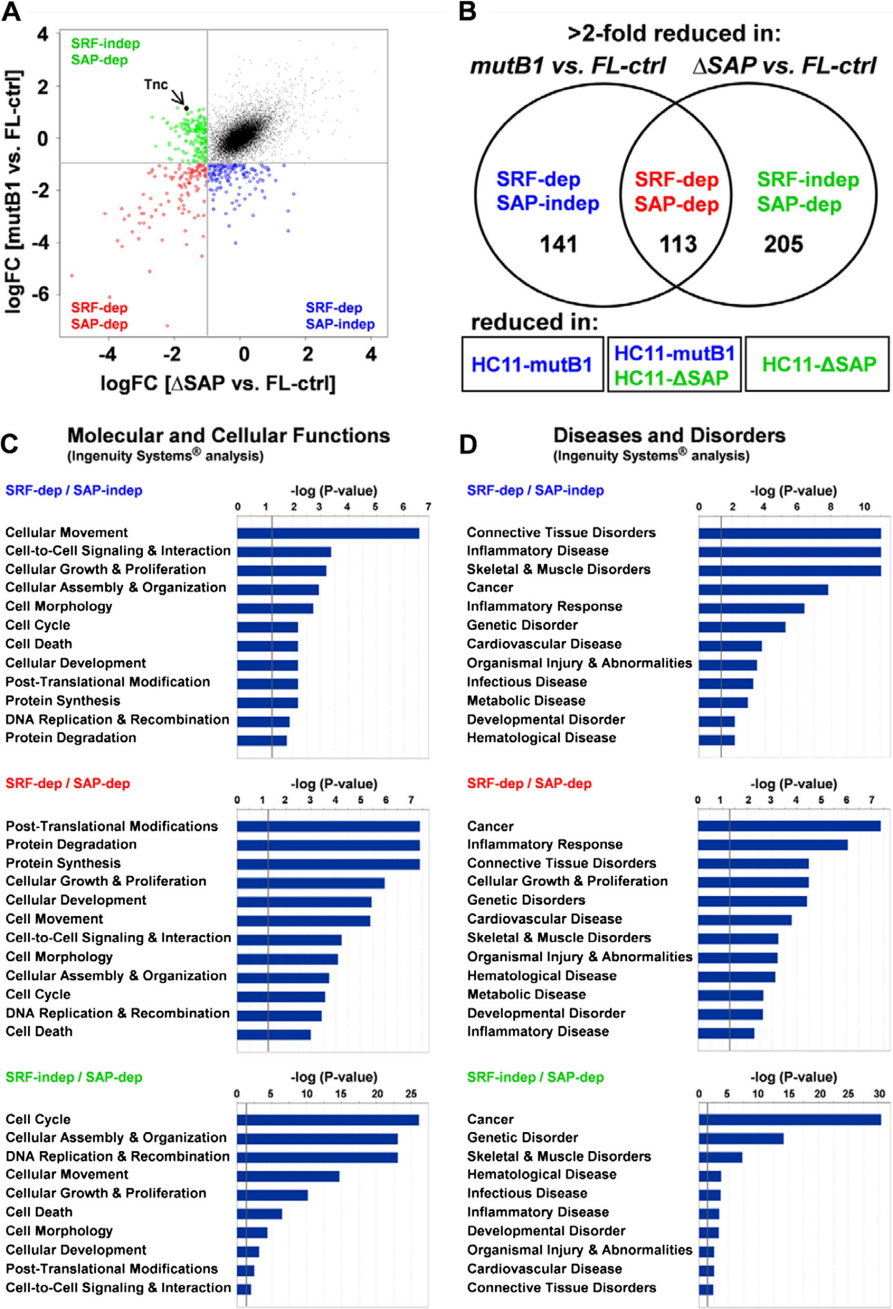
**Screen for SAP-dependent Mkl1 target genes and their implication in cancer. (A)** Scatter plot and **(B)** Venn diagram representing classification of Mkl1 target genes into three groups: SRF-dependent/SAP-independent (blue), SRF-dependent/SAP-dependent (red) and SRF-independent/SAP-dependent (green). The scatter plot **(A)** represents the log fold change (logFC) in gene expression in HC11-∆SAP versus HC11-FL control cells (x-axis; ∆SAP vs. FL) and between HC11-mutB1 versus HC11-FL control cells (y-axis; mutB1 vs. FL). Each dot represents a probeset, and the one for tenascin-C is highlighted (Tnc). The vertical and horizontal lines in the chart denote the 2-fold change cutoff (logFC = -1). The Venn diagram **(B)** represents the number of probesets for transcripts, which are more than 2-fold reduced in either HC11-mutB1 or HC11-ΔSAP cells when compared to HC11-FL control cells. Boxes below the Venn diagram indicate the cell strains that have reduced levels of the respective transcripts. **(C, D)** Functional analysis for the three Mkl1-regulated gene sets performed using the IPA software. The high-level functional **(C)** and disease **(D)** categories are displayed along the x-axis of each bar chart. The y-axis displays the –log of the P-value determined by right-tailed Fisher’s exact test. The P-value is a measure of the likelihood that the association between a set of genes in each dataset and a related function or disease is due to random association. The grey vertical line denotes the cutoff for significance (P = 0.05; -logP = 1.3).

We devised a screening method to identify genes co-regulated with tenascin-C by Mkl1 in a SAP domain-dependent manner without involvement of SRF. For this purpose, we used HC11 mammary epithelial cells that react strongly to the overexpression of Mkl1 with induction of tenascin-C expression [13]. We compared three HC11 strains that either overexpress the C-terminal red fluorescent protein (RFP)-tagged full length Mkl1 (HC11-FL), Mkl1-RFP with a mutated SRF-interaction site (HC11-mutB1) or Mkl1-RFP with a deletion of the SAP domain (HC11-ΔSAP). None of the three Mkl1 variants appear to be toxic to the cells, as we have not observed any changes in viability or cell morphology. HC11-FL cells were shown to overexpress Mkl1 7.1-fold above the endogenous Mkl1 present in parental HC11 cells [13], and were used as control cells in our study. All cell strains were FACS sorted to express similar levels of Mkl1-RFP proteins. These cells were used for transcript profiling and gene lists of interest were established as shown in Figure 1A, B. A scatter plot (Figure 1A) of all transcripts expressed in HC11-mutB1 versus HC11-FL control cells (y-axis) and all transcripts expressed in HC11-ΔSAP versus HC11-FL control cells (x-axis) shows that a large majority of transcripts does not differ significantly between the three cell strains (log fold change (FC) ≈ 0; black dots). Setting the threshold to a 2-fold reduction (logFC = -1; grey lines), three gene sets can be distinguished: 1) blue dots represent genes that are lower in HC11-mutB1 than in HC11-FL control cells, but are unaffected in HC11-ΔSAP cells, thus representing typical SRF/Mkl1 target genes; 2) green dots represent genes that are lower in HC11-ΔSAP than in HC11-FL control cells, but are unaffected in HC11-mutB1 cells (this gene set includes tenascin-C); and 3) red dots indicate genes with reduced expression in both HC11-mutB1 and HC11-ΔSAP cells compared to HC11-FL control cells. Thus, this approach enabled us to form three gene sets that were distinct from the large majority of transcripts and were dependent for expression on the B1 site of Mkl1, the SAP domain, or both. The three groups presented by a Venn diagram (Figure 1B) contain 141 probesets for transcripts that depended on the function of the B1 site but not the SAP domain for their induction, 113 probesets for transcripts that depended on both of these Mkl1 domains and a third group of 205 probesets for transcripts co-regulated with tenascin-C that did not require an interaction of Mkl1 with SRF but depended on the SAP domain for induction (complete probeset lists and annotations are found in Additional file 1: Table S1, Additional file 2: Table S2 and Additional file 3: Table S3). This analysis revealed that the SAP-dependent mechanism of tenascin-C regulation by Mkl1 is shared by a large cohort of genes. Below the Venn diagram, we indicated which cells were deficient in the respective transcripts. Thus, the typical SRF/Mkl1 target genes are reduced in HC11-mutB1 cells, while the SRF-independent/SAP-dependent genes are reduced in HC11-ΔSAP cells. The intermediate group that requires both Mkl1 activities is reduced in both the HC11-mutB1 and HC11-ΔSAP cells.

### The SAP-dependent Mkl1 target genes are implicated in cancer

Functional analysis of the three gene lists using the IPA software revealed different molecular and cellular functions (Figure 1C) and different disease associations (Figure 1D) for the three types of gene signatures. Thus, the SRF-dependent/SAP-independent signature implicated a function of these genes in cellular movement and the linked diseases included connective tissue disorders, inflammatory disease and skeletal and muscle disorders, which are the main features known to be regulated by SRF/Mkl1 interaction [23-25]. The SRF-dependent/SAP-dependent group of genes includes as major functions post-translational modification, protein degradation and protein synthesis, and the top disease association is cancer. Finally, the genes of the SRF-independent/SAP-dependent group were associated with extremely high significance with cell cycle and cancer (-logP ≥ 25 and ≥ 30, respectively), while the SRF/Mkl1 target genes were associated with the same two categories at low significance only (-logP ≥ 2 and ≥ 7, respectively). These data imply that SAP-dependent induction of transcription by Mkl1 may counteract the known differentiation-promoting effect of SRF/Mkl1-induced transcription. A list of SAP-dependent genes with published cancer-related functions, whose transcripts were downregulated more than 3-fold in HC11-ΔSAP compared to HC11-FL control cells, is presented in Table 1. To confirm that these transcripts are indeed differentially expressed in the different HC11 cell strains, qRT-PCR analysis was performed using cDNA from three different batches of the respective HC11 strains. Differences in gene expression between HC11-ΔSAP and control cells are presented in Table 1 and in more detail in Additional file 4: Figure S1. The qRT-PCR results agreed with the data obtained by transcript profiling. We also tested the SAP-dependent gene expression in the HC11 strains when grown in the presence of serum. It is interesting to note that in the presence of 3% FCS, these transcripts remained strongly reduced in HC11-ΔSAP compared to control cells (Table 1). Thus, the induction of these genes seems to depend mainly on whether the SAP domain is present in the transfected Mkl1 construct.

**Table 1:** SAP-dependent Mkl1 target genes

**Gene**	**Description**	**Fold Reduction in HC11-∆SAP vs. HC11-FL cells**			**Functions**
		**Microarrays in 0.03% FCS**	**qRT-PCR in 0.03% FCS**	**qRT-PCR in 3% FCS**	
**SRF-independent genes**					
Tnc	Tenascin C, ECM protein	3.07***	3.50***	26.34***	Cell adhesion, cell migration, wound healing and tissue remodeling, cancer cell invasion and metastasis [10]
Anln	Anillin, actin binding protein	3.10***	1.93***	1.38**	Cell cycle regulation [26], cell motility and cancer progression [26-28]
Nox4	NADPH oxidase 4	3.31***	94.19**	332.70***	Cell growth, differentiation and migration [29], tumor angiogenesis [30]
Adamts16	Metallopeptidase, ECM protein	3.63***	5.70***	14.84**	Cell growth and motility [31], role in arthritis [32] and cancer [31]
Krt5	Keratin 5, intermediate filament protein	3.73***	2.74***	8.02***	Protein synthesis, epithelial cell growth and differentiation [33,34]
p15 (PAF)	2810417H13Rik, PCNA-associated factor	3.91***	1.89***	1.34***	DNA repair and cell cycle regulation, cell survival and proliferation, tumorigenesis [35-37], hematopoiesis [38]
Ass1	Argininosuccinate synthetase 1	4.23***	3.89**	2.72**	Regulation of nitric oxide production and cell viability [39,40]
Cd34	CD34 antigen, stem cell antigen	4.25***	10.61***	1.72***	Vessel development and function [41], tumor growth [42,43]
Wisp1	WNT1 inducible signaling pathway protein 1, ECM protein	4.41***	2.54**	4.06**	Cell proliferation and survival, ECM deposition and turnover, EMT, tumorigenesis, tissue remodeling [44]
Mcm6	Minichromosome maintenance complex component 6	4.42***	2.83***	1.30***	Cell cycle regulation [45]
Car12	Carbonic anyhydrase 12	4.58***	16.11***	26.07**	Cell survival under hypoxic conditions, tumor-associated cell migration and invasion [46,47]
Htatip2	Hyaluronectin, TIP30, transcriptional regulator	5.89***	548.59***	245.27***	Regulation of apoptosis [48], tumor growth and metastasis [49]
Kif26b	Kinesin family member 26B	6.33***	8.36***	61.22***	Regulation of adhesion and cell polarity in kidney development [50]
**SRF-dependent genes**					
Lox	Lysyl oxidase, ECM protein	4.61***	4.70**	12.04***	ECM turnover, connective tissue remodeling and repair, tumor progression and metastasis [51,52]
Mmp12	Matrix metallopeptidase 12, metalloelastase	12.01***	23.49***	4.90**	ECM degradation in tissue remodeling [49] and tumorigenesis [53]
Mmp3	Matrix metallopeptidase 3, stromelysin-1	15.64***	14.70***	2.08**	ECM degradation in tissue remodeling [49] and tumorigenesis [54]

*Abbreviations:* ECM extracellular matrix protein, PCNA proliferating cell nuclear antigen, EMT epithelial-to-mesenchymal transition.

***P < 0.001, **P < 0.01, Student’s *t* test.

In addition, we monitored changes in the expression of some of the SRF-independent/SAP-dependent Mkl1 targets on a protein level. In agreement with the changes seen at the transcript level, we confirmed the reduction of tenascin-C, Wisp1 and Nox4 proteins in cells overexpressing the ΔSAP-Mkl1 construct compared to the HC11-FL control and HC11-mutB1 cells (Additional file 4: Figure S2). Using zymography, we found that Mmp2, a gene that was not affected by Mkl1 overexpression at the transcript level was highly expressed in all three cell strains, whereas Mmp3 and/or 12, which belonged to the SRF-dependent/SAP-dependent gene set, were almost completely lacking in HC11-mutB1 as well as HC11-ΔSAP cells, corresponding to the data obtained by transcript profiling.

### SRF-independent/SAP-dependent transcripts represent direct Mkl1 target genes

**Figure 2:**
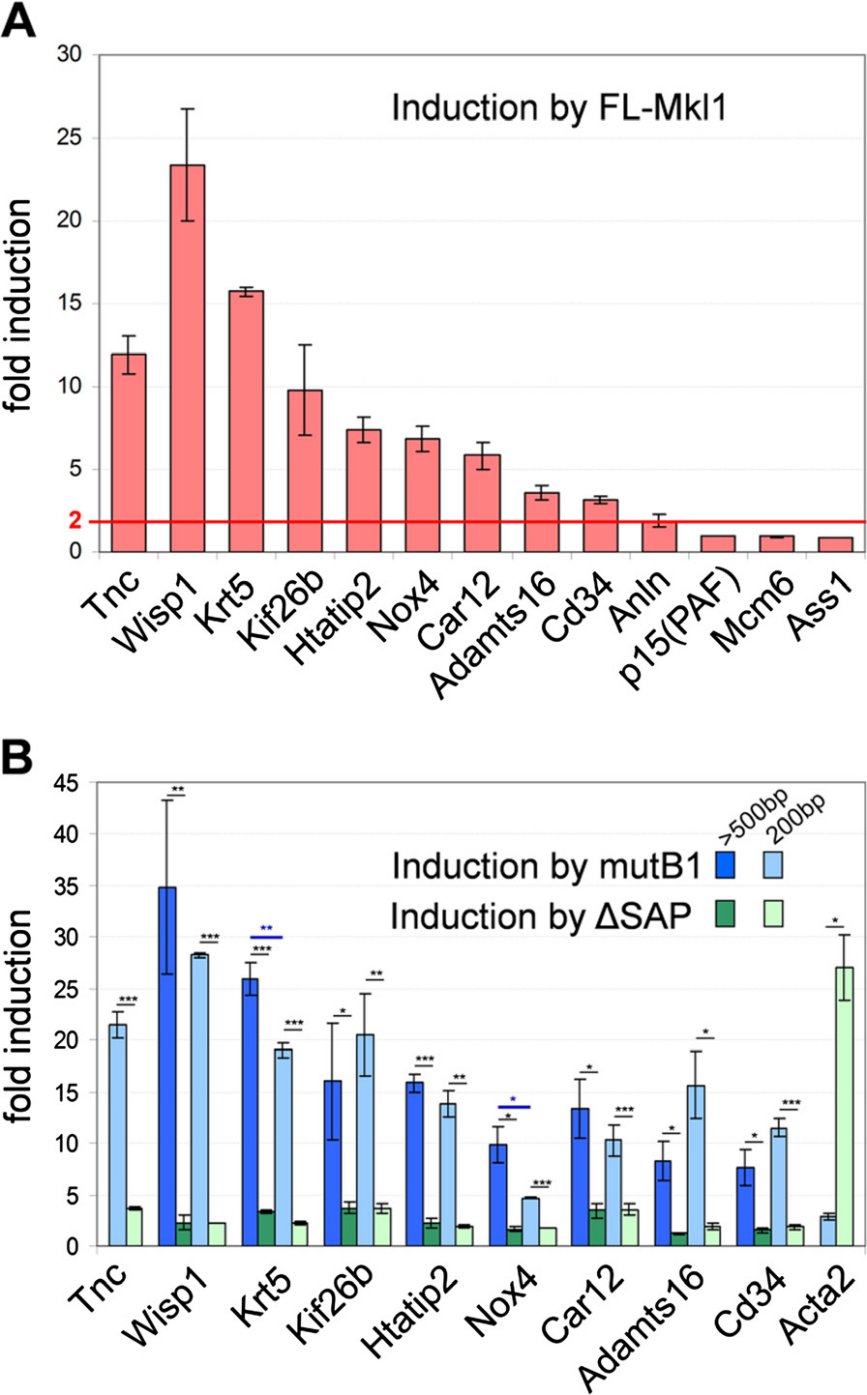
**SRF-independent/SAP-dependent transcripts represent direct Mkl1 target genes requiring the SAP domain of Mkl1 to induce transcription from their proximal promoter. (A)** The indicated promoter constructs that contained at least 500 bp upstream of the transcription start site (TSS) and were linked to the secreted alkaline phosphatase (SEAP) reporter gene, were cotransfected in HC11 cells together with an inactive Mkl1 devoid of the transactivation domain [13] or the FL-Mkl1 construct. SEAP activity is expressed as fold induction above the level obtained with the inactive Mkl1. In addition to Tnc, for 8 out of the 12 new promoters tested, induction greater than 2-fold (indicated by the red line) was obtained. Values are means ± SEM from three to seven independent experiments. **(B)** HC11 cells were cotransfected with the indicated promoter constructs that were either > 500 bp or shortened to 200 bp upstream of the TSS, and with vectors encoding the indicated mutant Mkl1 constructs. SEAP activity is normalized and expressed as in **(A)**. Means ± SEM from at least three independent experiments and significant differences between either mutB1- and ΔSAP-Mkl1-transactivated promoter constructs or between the longer and shorter promoter constructs transactivated by mutB1-Mkl1, ***P < 0.001, **P < 0.01, *P < 0.05 are shown.

Since we have previously shown that the SAP domain of Mkl1 interacts with the proximal promoter of tenascin-C to induce its transcription [13], we tested whether this was also the case for other transcripts of the same group. The promoters of the SRF-independent/SAP-dependent genes listed in Table 1 encompassing at least 500 bp upstream of the transcription start site (TSS) were fused to the secreted alkaline phosphatase (SEAP) reporter gene of pSEAP2-Basic. We tested the induction of each promoter-reporter construct by co-transfection with FL-Mkl1 (Figure 2A). This revealed that the majority of the new promoters tested (8 out of 12) were induced at least 2-fold by Mkl1 in comparison to co-transfection with an inactive Mkl1 devoid of the transactivation domain, indicating that these are indeed direct Mkl1 target genes. The promoter constructs that did not respond to Mkl1 overexpression may represent genes that are indirectly regulated by Mkl1, or the relevant promoter regions were not contained in the constructs tested. Next, we investigated whether the induction was SAP-dependent and SRF-independent by comparing the reporter activity after co-transfection with mutB1- versus ΔSAP-Mkl1 variants (Figure 2B). Indeed, the promoter-reporter constructs induced by FL-Mkl1 were also strongly induced by mutB1-Mkl1, but not by ΔSAP-Mkl1. In contrast, the promoter construct for Acta2, a gene from the SRF-dependent/SAP-independent gene set was strongly induced by ΔSAP-Mkl1 but not by mutB1-Mkl1, as expected for a typical SRF/Mkl1 target gene [18,55,56]. All promoters that revealed SAP-dependency were shortened to 200 bp upstream of the TSS to test whether this was sufficient to relay the Mkl1 response, as it has been seen previously for tenascin-C [13]. With the exception of Krt5 and Nox4, for which some activity was lost by shortening the promoters, the 200 bp proximal promoters of all other genes tested were induced equally well as the longer constructs (Figure 2B). Thus, we conclude that there are many genes that are regulated similarly as tenascin-C requiring the SAP domain of Mkl1 to induce transcription from their proximal promoter.

### The different HC11 cell strains proliferate at different rates and show distinct migration behaviors

**Figure 3:**
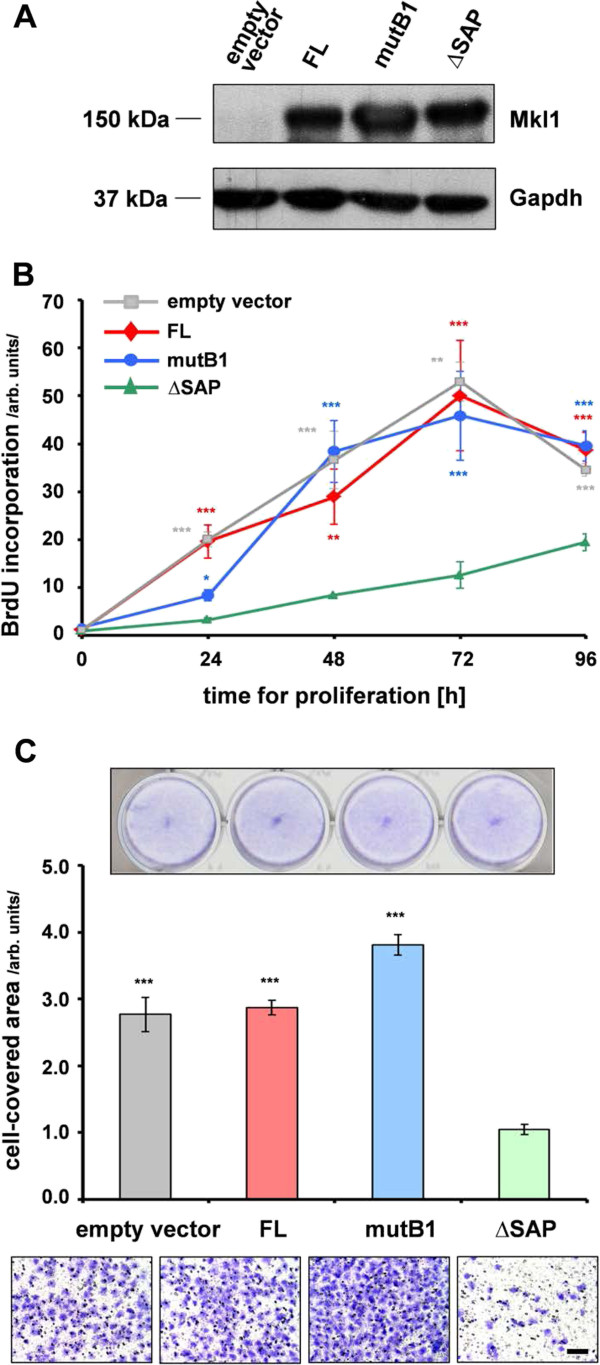
**The different HC11 cell strains proliferate at different rates and show distinct migration behaviors. (A)** Immunoblot with mAb65F13 of Mkl1 proteins in whole-cell extracts from the empty vector-, FL-, mutB1- or ΔSAP-transfected HC11 strains. Anti-Gapdh served as loading control. Endogenous Mkl1 protein was below the detection limit in empty vector cells. **(B)** SAP-dependent proliferation of HC11 mammary epithelial cells. Proliferation rates of the four HC11 cell strains were assessed by BrdU incorporation into newly synthesized DNA immediately after plating (0 h) as well as at 24, 48, 72 and 96 h. Means ± SD from three independent experiments and significant differences to the HC11-ΔSAP cells, ***P < 0.001, **P < 0.01, *P < 0.05 are shown. **(C)** SAP-dependent migration of HC11 mammary epithelial cells. Cell migration of the four HC11 strains was evaluated by Transwell migration assay using filters with 8 μm pore size. Quantification of the cell migration was measured by the area on the lower side of the filter covered with cells. Above the bar graph, a photo of fixed and stained cells seeded in parallel in a 24-well plate is shown as a seeding control, and representative photos of fixed and stained cells of each of the cell strains that have migrated to the lower side of the filter, are shown below (bar, 200 μm). Data and statistical significance are expressed as in **(B)**.

Next, we tested whether the differential gene expression seen in the different HC11 strains overexpressing either FL-, mutB1- or ΔSAP-Mkl1 constructs have functional consequences on their behavior. Since most of the SAP-dependent transcripts are proposed to have a function in cancer, we decided to analyze two main functions important for cancer progression: proliferation and cell migration. An approximately equal overexpression of the different Mkl1 protein variants in the HC11 cell lines was confirmed by Western blot analysis (Figure 3A). An HC11 cell strain stably transfected with an empty vector [13] expressing only endogenous Mkl1 (below the detection limit in Figure 3A) was also included in these studies. The proliferation rates of the HC11 strains were analyzed using a 5-bromo-2′-deoxyuridine (BrdU) incorporation assay. The incorporated BrdU was measured immediately after plating (0 h) as well as at 24, 48, 72 and 96 h. Compared to empty vector-, FL- or mutB1-transfected HC11 strains, there was a significant decrease in BrdU uptake into newly synthesized DNA in HC11-ΔSAP cells over the entire time period tested (Figure 3B). To investigate cell motility, we used a transfilter migration assay. Similarly to the effect on cellular proliferation, the expression of ΔSAP-Mkl1 significantly inhibited HC11 cell migration by 2.7-fold compared to endogenous or full length Mkl1 expression, and more than 3.5-fold compared to mutB1-Mkl1 expression (Figure 3C).

Thus, overexpression of FL-Mkl1 protein in HC11 cells did not affect their behavior. However, overexpression of ΔSAP-Mkl1 led to a significant reduction in the proliferative and migratory ability of HC11 epithelial cells, either through a dominant-negative effect of ΔSAP-Mkl1 on SRF-mediated action and/or a positive impact of the SAP-dependent Mkl1 target genes on these functions important for cancer progression.

### SAP-dependent Mkl1 target genes are mechanoresponsive

**Figure 4:**
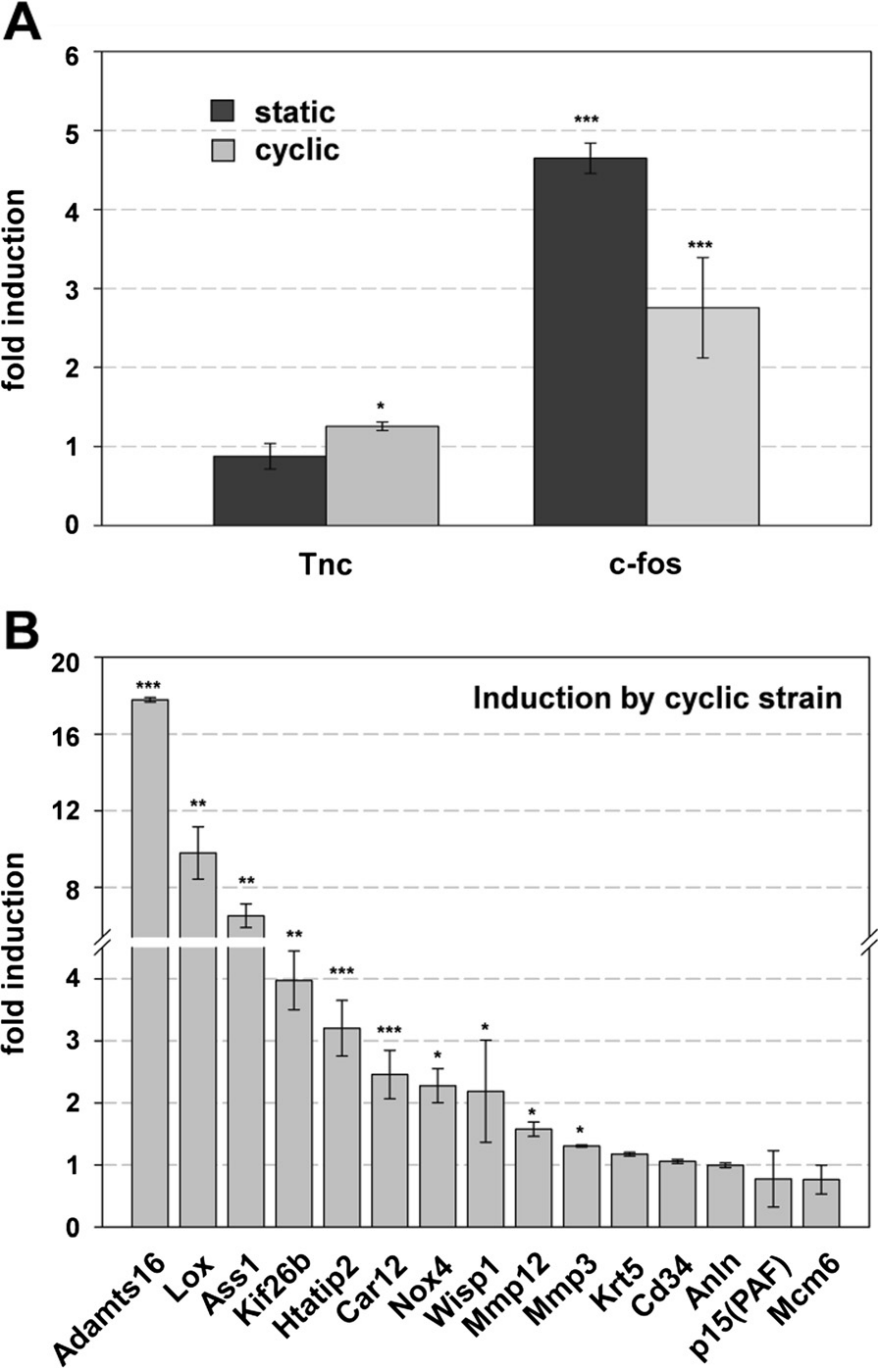
**SAP-dependent Mkl1 target genes are mechanoresponsive. (A)** Effect of static (20%) and cyclic (15%, 0.3 Hz) strain on Tnc and c-fos mRNA levels. HC11 cells were cultured on either growth factor reduced matrigel matrix- or fibronectin-coated silicone membranes in 0.03% serum-containing medium for 24 h before applying static or cyclic strain for 1 h. Cells cultured under the same conditions and not exposed to mechanical stimulation were used as a resting control. The two types of coating gave identical results under the indicated experimental conditions. Total RNA was extracted and qRT-PCR was performed for Tnc and c-fos mRNA levels. Values normalized to Gapdh are expressed relative to the values of resting cells. Data represent means ± SD from three independent experiments. Significant differences to the resting control, ***P < 0.001, **P < 0.01, *P < 0.05. **(B)** SAP-dependent genes respond to cyclic strain. HC11 cells were stretched and mRNA analyses were performed as described in **(A)**. Data and statistical significance are expressed as in **(A)**.

We have previously found that the SAP-dependent induction of tenascin-C was triggered by applying mechanical strain to fibroblasts. Mammary epithelial cells are also exposed to mechanical strains, both during normal development, pregnancy and lactation, as well as under pathological conditions such as in cancer. Therefore, we tested whether tenascin-C and other members of the SAP-dependent Mkl1-induced gene set are mechanoresponsive in HC11 cells. We tested two paradigms: 1) static strain that was shown to induce c-fos, a very prominent mechanoresponsive gene in HC11 cells [57] that we used as a control, and 2) cyclic strain. While we were able to confirm induction of c-fos by applying static strain at 20% for 1 h, there was no induction of tenascin-C under these conditions compared to cells at rest (Figure 4A). However, using 15% cyclic strain at a frequency of 0.3 Hz for 1 h, we found that not only the control gene c-fos but 11 out of 16 SAP-dependent genes, including tenascin-C were significantly upregulated above the expression levels obtained in resting cells (Figure 4). Even though significant, the induction of tenascin-C was minimal (Figure 4A) compared to 18-fold upregulation for Adamts16 or 10-fold upregulation for Lox (Figure 4B), both of which are enzymes involved in extracellular matrix (ECM) remodeling and cancer progression [31,58]. Being mechanoresponsive, the SAP-dependent Mkl1 target genes might be activated in stiff tumor tissue, which further confirms their relation with cancer.

### The SRF-independent/SAP-dependent genes represent a bad prognostic signature for breast cancer patients

**Figure 5:**
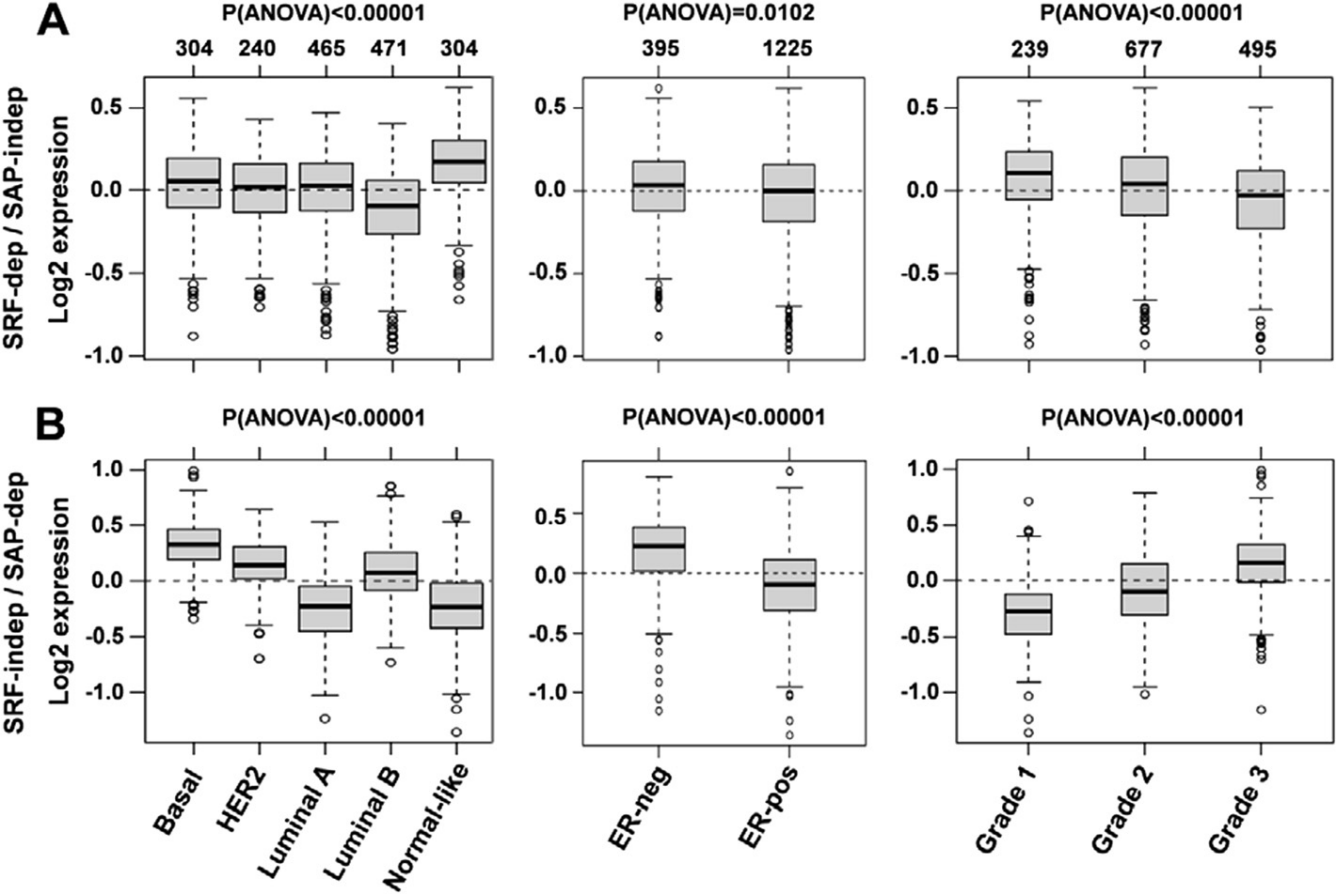
**SAP-dependent Mkl1 target genes are associated with typical high-proliferative poor outcome classes in breast cancer.** The expression levels for the SRF-dependent/SAP-independent **(A)** and SRF-independent/SAP-dependent **(B)** gene sets are analyzed across the 1881-sample breast cancer data set stratified according to PAM50 subtypes (left panels), estrogen receptor (ER)-status (middle panels) and histological grade (right panels), and represented by box plots using the GOBO bioinformatics tool. The number of tumors in each breast cancer subtype and the significant difference in gene expression (P-value calculated using ANOVA) between them are shown above the box plots.

**Figure 6:**
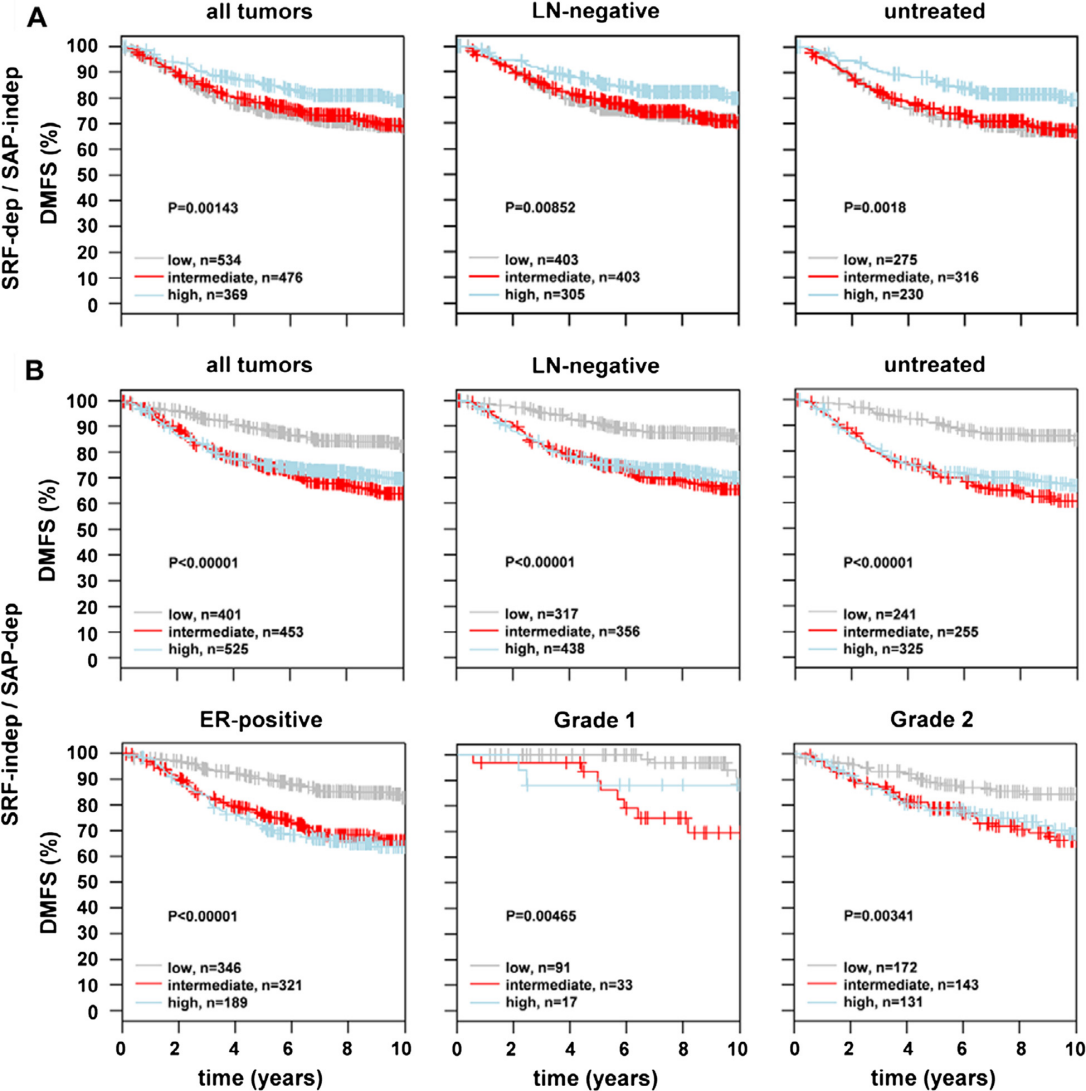
**The SRF-independent/SAP-dependent genes represent a bad prognostic signature for breast cancer patients.** Tumors in the 1881-sample breast cancer data set were stratified into three quantiles, low, intermediate and high, based on SRF-dependent/SAP-independent **(A)** or SRF-independent/SAP-dependent **(B)** gene expression. **(A)** Kaplan-Meier survival analysis using distant metastasis free survival (DMFS) as endpoint and 10-year censoring for all tumors (n = 1379; left panels), or in the subgroups of lymph node (LN)-negative (n = 1111; middle panels) and untreated tumors (n = 821; right panels) was performed using the GOBO bioinformatics tool, interrogating the group of SRF-dependent/SAP-independent target genes. P-value is calculated using log-rank test. **(B)** Kaplan-Meier survival analysis for tumors with expression of SRF-independent/SAP-dependent Mkl1 target genes was performed as in **(A)**. Association with clinical outcome was assessed in the subgroups of ER-positive (n = 856; left panel), Grade 1 (n = 141, middle panel) and Grade 2 (n = 446; right panel) tumors in addition to all tumors and the subgroups used in **(A)**. P-value is calculated using log-rank test.

**Figure 7:**
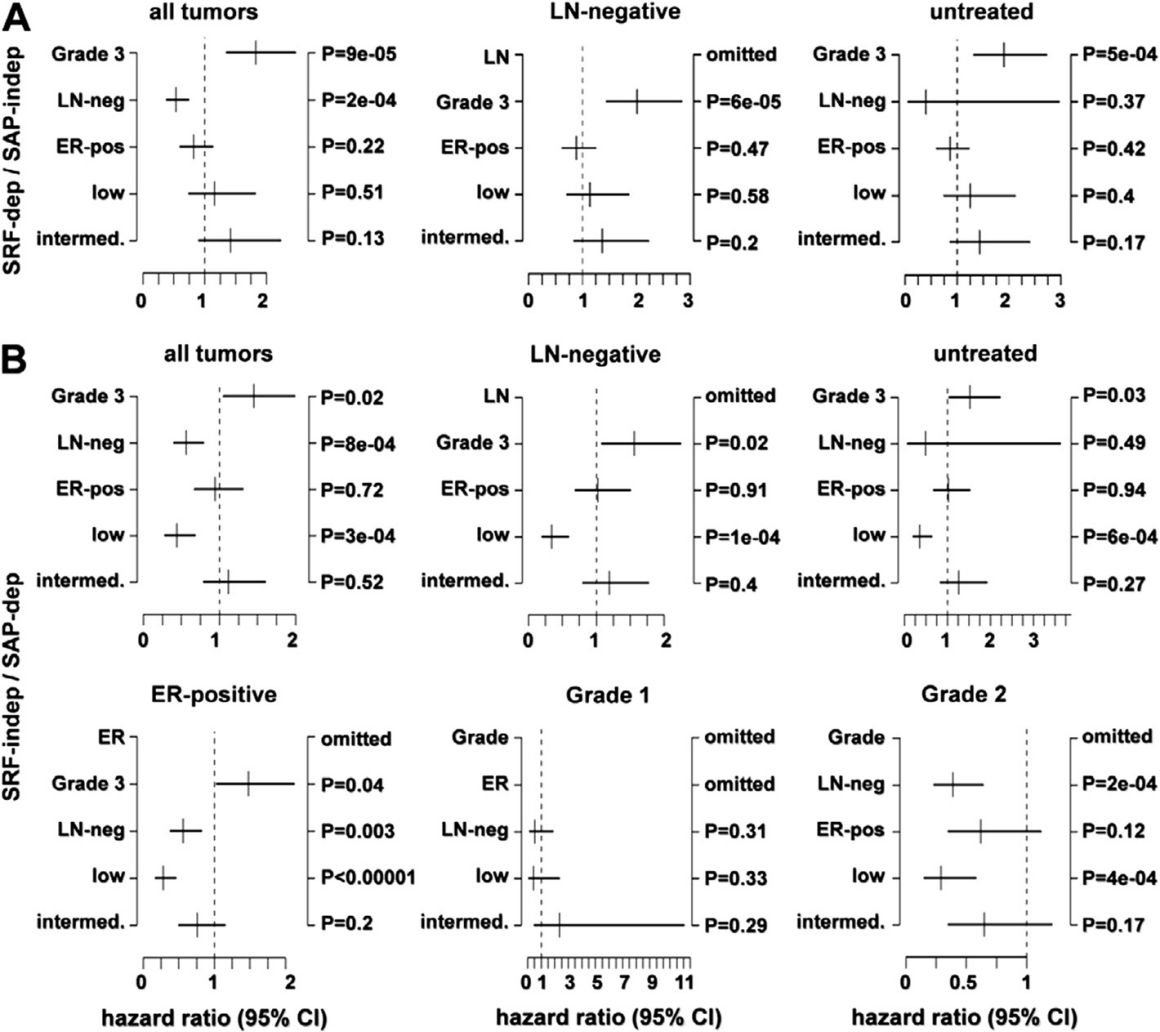
**Elevated expression of SAP-dependent Mkl1 target genes is a poor prognosis factor in breast cancer independent of histological grade.** Multivariate analysis supporting the Kaplan-Maier survival analysis (shown in Figure 6) for the SRF-dependent/SAP-independent **(A)** and SRF-independent/SAP-dependent **(B)** gene sets, was performed using the GOBO bioinformatics tool. The analysis was executed for all tumors (n = 1379) and in the subgroups of LN-negative (n = 1111) and untreated tumors (n = 821) **(A, B)**, as well as in the subgroups of ER-positive (n = 856), Grade 1 (n = 141) and Grade 2 (n = 446) tumors **(B)**, using LN-status, ER-status, and stratified histological grade (histological grade 1 and 2 vs. 3) as covariates, DMFS as endpoint and 10-year censoring. The hazard ratio and the 95% confidence interval (CI) are plotted for each of these covariates. Specified covariates may be omitted in certain comparisons, e.g. ER-status is omitted when analyzing ER-positive tumors only, or when not all of the investigated cases have clinical follow-up or clinical information for the specified covariate.

In order to investigate whether the SRF-independent/SAP-dependent genes were prognostic of accelerated cancer progression in human patients, we used the bioinformatics tool Gene expression-based Outcome for Breast cancer Online (GOBO) that allowed us to investigate a breast tumor data set containing 1881 samples analyzed by Affymetrix Human Genome U133A arrays. GOBO is designed to assess gene expression levels and association with outcome of single genes or gene sets in multiple subgroups of this breast cancer data set [59]. Here, we analyzed two sets of genes, namely the SRF/Mkl1-induced gene set (SRF-dependent/SAP-independent) and the SAP-dependent gene set (SRF-independent/SAP-dependent) containing tenascin-C. The analysis was performed across tumor samples stratified according to PAM50 subtypes [60], estrogen receptor (ER)-status and histological grade. In contrast to the SRF/Mkl1 target genes that were predominantly associated with tumors classified as normal-like and with lower histological grades (1 and 2) (Figure 5A), elevated expression of SAP-dependent genes was associated with extremely high significance (P < 0.00001) with typical high-proliferative poor outcome classes in breast cancer, such as basal-like, HER2-enriched, luminal B, ER-negative and histological grade 3 tumors (Figure 5B). Next, a functional correlation analysis to find a possible interconnection between the SAP-dependent Mkl1 target genes was performed using the GOBO tool (Additional file 4: Figure S3). This analysis explores the correlation of expression of individual genes in our gene sets with eight different co-expressed gene modules emulating breast cancer-specific as well as general tumor biological processes [61]. Interestingly, whereas the gene set of SRF/Mkl1 targets did not show a significant correlation with any of these modules, the genes in the SAP-dependent gene set were correlated with a very high significance (P < 0.00001) with two proliferation modules – mitotic checkpoint and mitotic progression. Both modules contain genes related to central mitotic processes involved in either the regulation of the M-phase and the mitotic checkpoint or in carrying out the M-phase. Finally, the association of our gene sets with outcome using distant metastasis free survival (DMFS) as an endpoint and 10-year censoring was analyzed. The survival analysis was performed in all tumors for which DMFS follow-up is available (1379 cases), as well as in 21 groups that were stratified based on gene expression subtypes (PAM50 classifier), ER-status, lymph node (LN)-status, histological grade, and treatment status. Samples in the whole cancer data set (1881 patients) were stratified into three quantiles, low, intermediate and high, based on SRF-dependent/SAP-independent or SRF-independent/SAP-dependent gene expression. Interestingly, high expression of SRF/Mkl1-induced genes was associated with a better clinical outcome for all tumors, as well as for LN-negative and untreated tumors compared to low and intermediate expression of these genes (Figure 6A). In contrast, both high and intermediate expression of the SAP-dependent genes was associated with bad clinical outcome in all tumors, and particularly in LN-negative, systemically untreated, ER-positive, Grade 1 and 2 tumors (Figure 6B). Similar results were obtained for the typical breast cancer gene CCNB1 by Ringnér et al. [59]. The Kaplan-Meier survival analyses were supported by the corresponding multivariate analyses (Figure 7A, B). The hazard ratio for the variate Grade shows statistical significance, proving that the influence of high SAP-dependent gene expression on patient survival is independent of tumor grade. Among all tumors for which DMFS data are available, a hazard ratio of 0.44 (95% CI = 0.28-0.68; P = 0.0003) for the low SRF-independent/SAP-dependent tercile was detected compared to the high SRF-independent/SAP-dependent tercile (Figure 7B, all tumors). This indicates that patients with tumors expressing high levels of the SAP-dependent genes are more than twice as likely to develop metastatic disease. Similar hazard ratios, in the range of 0.28-0.44 for the low tercile compared to the high tercile were also detected among subgroups of untreated, LN-negative, ER-positive, Grade 1 and 2 tumors (Figure 7B). Thus, the association of high SRF-independent/SAP-dependent gene expression with reduced DMFS among patients not receiving adjuvant therapy, as well as among LN-negative, ER-positive, Grade 1 and 2 patients indicates that increased expression of the SAP-dependent Mkl1 target genes plays a significant role in the natural metastatic progression of non-aggressive towards highly aggressive breast cancer in human patients.

## Discussion

Given the heterogeneity of mutations in tumor cells, it becomes increasingly clear that not only individual genes but pathways govern the course of tumorigenesis and cancer progression [62]. We have recently shown that induction of tenascin-C by cyclic mechanical strain required the action of the potential DNA-binding SAP domain of Mkl1 independently of an interaction of Mkl1 with SRF [13]. Now, we report a screen for genes co-regulated with tenascin-C by the same SAP-dependent and SRF-independent mechanism in mammary epithelial cells. This screen reveals a set of SAP domain-dependent Mkl1 target genes with a strong implication in cell proliferation, cell motility and cancer.

**Figure 8:**
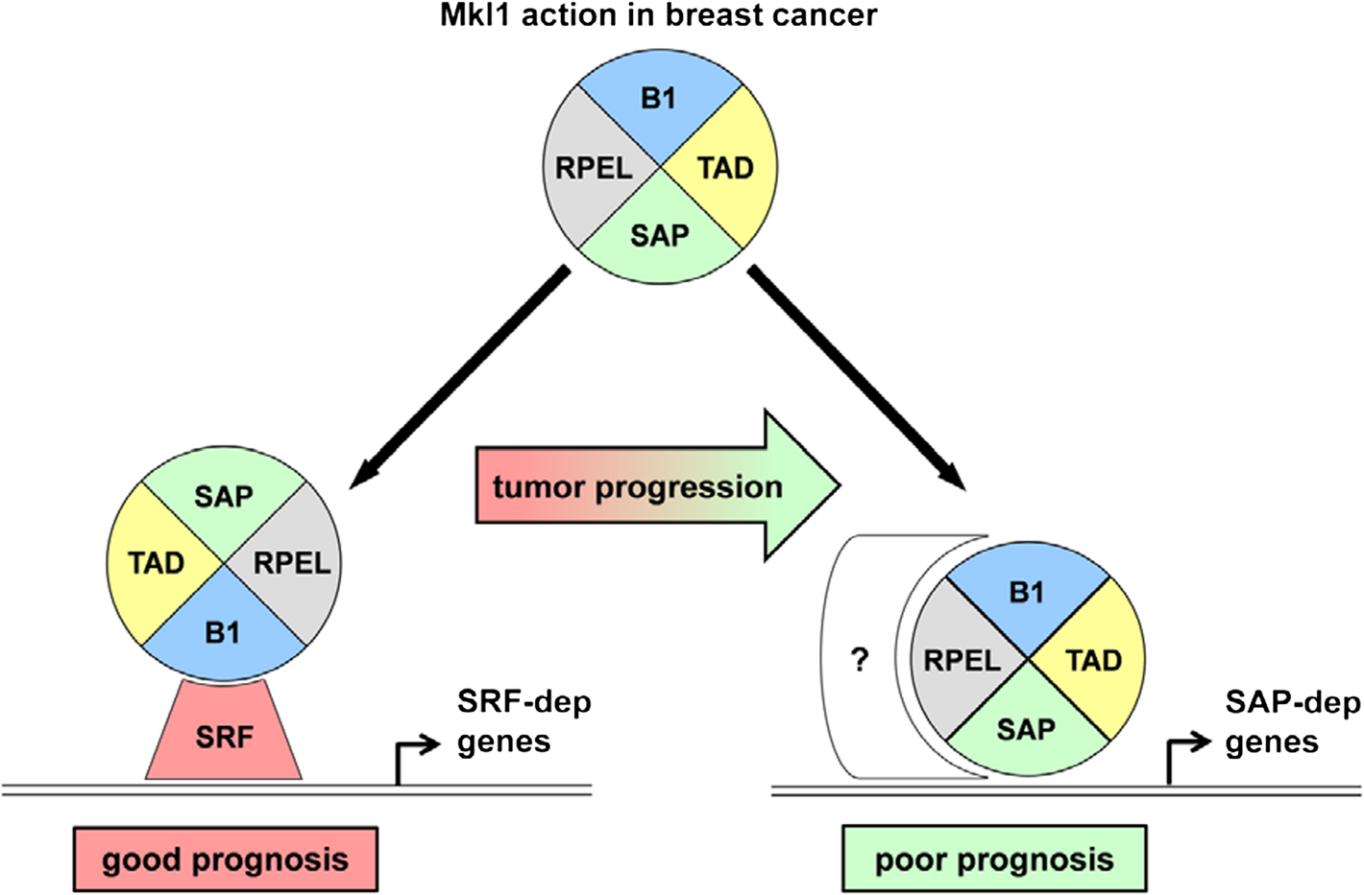
**Schematic representation of the Mkl1 action in breast cancer.** A circular Mkl1 model is depicted with four of its domains: RPEL, actin binding motifs with RPxxxEL core consensus; B1, basic domain involved in SRF-binding; SAP, homology domain found in the nuclear proteins SAF-A/B, Acinus, PIAS; TAD, transactivation domain. Serum response factor is drawn as a red shape and putative unidentified DNA-binding proteins as white shape with a question mark. Mkl1 exerts two distinct modes of action: one of them is through the B1 domain required for serum response factor (SRF)-binding activity and induction of SRF/Mkl1 target gene expression; the other one is strongly dependent on the SAP domain and triggers the expression of a specific set of pro-proliferative and pro-migratory genes that we called SAP-dependent Mkl1 target genes. High expression of SRF-dependent genes is associated with good clinical outcome for breast cancer patients, whereas elevated expression of SAP-dependent targets correlates with poor prognosis and indicates a significant role for these genes in breast cancer progression.

To date only a few studies have shown that Mkl1 is implicated in cancer-related processes (reviewed in [63]) and most of them have concentrated on the SRF/Mkl1 signaling for the induction of individual genes [64-67]. The first study reporting that depletion of Mkl1/2 proteins reduced motility, invasion and colonization of metastatic tumor cells in an experimental *in vivo* metastasis assay [64] was further supported by the discovery of the Mkl1-binding protein, suppressor of cancer cell invasion (SCAI), which inhibited SRF/Mkl1-mediated expression of β1 integrin [68]. Since then, several studies describing opposing biological effects for Mkl1 appeared. For instance, several antiproliferative SRF/Mkl1 target genes including mig6/errfi-1, a negative regulator of the EGFR-MAPK pathway, were identified [65], or the tumor suppressor gene Eplin-α was described as a direct target of the SRF/Mkl1 pathway [66]. Furthermore, expression of a constitutively active form of Mkl1 in oncogenic ras- or src-transformed rat intestinal epithelial cells injected into the spleen of nude mice significantly suppressed tumor formation and reduced liver metastases by rescuing the expression of the SRF/Mkl1 targets tropomyosin and caldesmon [67]. In line with these findings, we could show that high expression of SRF/Mkl1 target genes is associated with an improved clinical outcome in breast cancer patients. However, the opposite is the case for high expression of SAP-dependent Mkl1 target genes. These genes are associated with poor clinical outcome predominantly in less aggressive tumors such as LN-negative, ER-positive, Grade 1 and 2 tumors, which makes them valuable predictors of breast cancer progression. A scheme that depicts our model for Mkl1 action in breast cancer is presented in Figure 8. In this model Mkl1 is transactivating SRF-target genes in less aggressive tumors, while in the course of cancer progression and metastatic behavior Mkl1 is activating a new group of genes in a SAP-dependent manner either by direct interaction with the promoters of these genes or by interaction with additional DNA-binding factors.

Interestingly, in parental HC11 cells many of the genes that we found in the SAP-dependent gene set that foster cell proliferation and migration and may cause poor survival of breast cancer patients are also induced by mechanical strain. A recent study has demonstrated that inhibition of cell spreading due to a lack of matrix stiffness is overcome by externally applied stretch, suggesting that similar mechanotransduction mechanisms sense stiffness and stretch [69]. Tumor stroma is typically stiffer than normal stroma. In breast cancer, diseased tissue can be 10 times stiffer than normal breast [70,71]. It is known that abnormal ECM stiffness plays an important role in cancer progression [72,73], but the mechanisms by which stiffness influences cancer progression are still under investigation. If we assume that we have discovered a general reaction of mammary epithelial cells to mechanical strain, we envisage that epithelial cells in a stiff, mechanically dynamic tumor environment may react by inducing a SAP-dependent Mkl1 gene set that in turn affects tumor progression. Furthermore, the products of these genes, many of which are involved in ECM turnover and function, for example Lox [58], Mmps [74], Adamts16 [31] or Wisp1 [44] might themselves manipulate the tumor microenvironment, thereby influencing tumor cell survival by a positive tumorigenic feedback loop.

Finding how to switch the mode of action of Mkl1 between SRF transactivation versus its SAP-dependent transcriptional activity is a subject of ongoing research in our lab that in future may help with the development of new therapeutic interventions for breast cancer. Post-translational modifications such as sumoylation are known to influence Mkl1 transcriptional activity [75] and phosphorylation has been shown to influence interaction of Mkl1 with nuclear actin resulting in transcriptional changes [76,77]. Further characterization of these and other post-transcriptional changes of Mkl1 deserve special attention when trying to answer the above question.

## Conclusions

In the current study, we discovered a breast cancer-specific set of genes that is highly interesting as a prognostic marker and therapeutic target for several reasons. (1) The expression of this gene set is regulated by Mkl1 and its SAP domain and is independent of SRF. (2) The SAP-dependent, SRF-independent Mkl1signaling is triggered by mechanical strain and may thus be activated in stiff tumors with a high stromal content and high interstitial tissue pressure. (3) This gene set is composed of interesting members some of which represent novel candidates for playing a functional role in cancer and others that have already been implicated in cancer-related functions, as for example tenascin-C, a metastatic niche component important for lung colonization [8], or Lox as a gene mediating collagen crosslinking responsible for fibrosis-enhanced metastasis [58]. (4) The SAP-dependent Mkl1 target genes are associated with a poor clinical outcome in breast cancer patients, not receiving adjuvant therapy or having a cancer classified as non-aggressive such as LN-negative, ER-positive, Grade 1 or 2 tumors. This makes these genes potential valuable prognostic markers in selecting patients who may benefit from an immediate and/or more aggressive therapy.

## Methods

### Cell culture

Full length Mkl1 (FL-Mkl1) and the two Mkl1 mutants, mutB1-Mkl1 comprising alanine substitutions of four amino acids (underlined) in the B1 domain of Mkl1 (KKAKELKPKVKKLKYHQYIPPDQKQD) [78] and ∆SAP-Mkl1 with a deletion of the SAP domain [15], were constructed based on transcript variant 1 (GenBank accession number NM_153049) as previously described [13]. All Mkl1 variants were expressed as C-terminal RFP-tagged fusions. An empty vector expressing RFP alone was previously described [13].

HC11 mammary epithelial cells, kindly provided by Dr. N. Hynes (Basel, Switzerland), were grown in RPMI-1640 medium supplemented with 10% FCS, 5 μg/ml insulin (Sigma, Buchs, Switzerland) and 10 ng/ml epidermal growth factor (EGF; Invitrogen, Zug, Switzerland). In most of the experiments, the HC11 cells were starved in 0.03% FCS/RPMI without EGF. To obtain HC11 cells stably expressing FL-Mkl1-RFP (HC11-FL), mutB1-Mkl1-RFP (HC11-mutB1), ∆SAP-Mkl1-RFP (HC11-∆SAP) or RFP alone (HC11-empty vector), cells were transfected using FuGENE® 6 (Roche, Basel, Switzerland) and selected with Geneticin (1 mg/ml; Roche) for 14 days before fluorescence-activated cell sorting (FACS) of RFP-positive cells on a Vantage SE (Becton Dickinson, Basel, Switzerland). Cell viability of the four HC11 cell strains was assessed by the CellTiter-Blue viability assay (Promega, Duebendorf, Switzerland).

### Cell proliferation assay

Proliferation rates of the HC11 cell strains were determined using BrdU incorporation assay (Roche). After 24 h of starvation, cells were plated in triplicate on Black 96-well microtiter plates (PerkinElmer, Schwerzenbach, Switzerland) at 5 × 10^3^ cells/well in 3% FCS/RPMI and allowed to proliferate for 0, 24, 48, 72 and 96 h before labeling with BrdU for 2 h. BrdU incorporation into newly synthesized DNA was determined according to the manufacturer’s protocol using a Luminometer Mithras LB940 (Berthold Technologies, Regensdorf, Switzerland). Experimental values were normalized to the values of HC11-∆SAP cells at the time point 0. Data represent means ± SD from three independent experiments.

### Cell migration assay

Cell migration was assayed using transwell polycarbonate membrane inserts (6.5 mm; Corning, Amsterdam, The Netherlands) with 8 μm pores as described [79]. After 24 h of starvation, 5 × 10^4^ cells were plated in the top insert chamber with 100 μl serum-free RPMI. The lower chamber was filled with 600 μl 10% FCS/RPMI. Cells were allowed to migrate across the filter for 22 h at 37°C before fixation and crystal violet-staining. Images of duplicate inserts were acquired on a Nikon Eclipse E600 using 10× magnification and a color CCD camera. Migration was quantified by measuring the area covered by migrated cells using the Fiji distribution of ImageJ [80]. Data represent means ± SD from three independent experiments.

### Mechanical stimulation of cells

2 × 10^5^ HC11 cells/well were seeded in BioFlex® 6-well culture plates (Flexcell International, Hillsborough, NC, USA) coated with either growth factor reduced-Matrigel (BD Biosciences, Basel, Switzerland) or fibronectin [11]. Cultures were starved for 24 h before applying either equibiaxial cyclic strain (15%, 0.3 Hz) or static strain (20%) at 37°C for 1 h using Flexcell FX-4000 (Flexcell International). Cells cultured under the same conditions and not exposed to strain were used as a resting control. After mechanical stimulation, cells were lysed and total RNA was isolated using the RNeasy Mini Kit (Qiagen, Basel, Switzerland).

### Transcript profiling and bioinformatics analysis

HC11 cell strains stably expressing Mkl1 variants were starved for 48 h before total RNA was extracted, converted into labeled cDNA and hybridized to Affymetrix GeneChip Mouse Gene 1.0 ST arrays. RMA-normalized expression values were calculated with the Affy package from Bioconductor 2.4 [81], and differentially expressed genes were identified using moderated *t*-statistics calculated with the empirical Bayes method as implemented in the Bioconductor limma package [82]. To be considered as differentially expressed between HC11-FL and HC11-mutB1 or HC11-∆SAP cells, genes had to pass the filters: adjusted P-value ≤ 0.01 (with Benjamin-Hochberg false discovery correction), a minimum absolute linear fold change difference of 2.0 and a minimum average expression value of 4.0 (log2). Microarray data files are available from the Gene Expression Omnibus (GEO; http://www.ncbi.nlm.nih.gov/geo/), accession number GSE44907. Using the above parameters, gene lists of the two contrasts (mutB1/FL and ∆SAP/FL) were compared resulting in the formation of three gene groups: SRF-dependent/SAP-independent, SRF-dependent/SAP-dependent and SRF-independent/SAP-dependent. The three gene sets were analyzed using the bioinformatics softwares: 1) IPA (Ingenuity® Systems; http://www.ingenuity.com); and 2) GOBO (http://co.bmc.lu.se/gobo) [59]. In order to use the latter tool, Affymetrix GeneChip Mouse Gene 1.0 ST IDs were mapped to Affymetrix Human Genome U133A IDs using Biomart for Ensembl build 66. The module “Gene Set Analysis Tumors” was used to investigate the expression pattern and to perform survival and functional correlation analyses for the SRF-dependent/SAP-independent and SRF-independent/SAP-dependent gene sets across 1881 breast cancers characterized by Affymetrix Human Genome U133A arrays.

### RNA analyses by qRT-PCR

Total RNA was isolated from HC11 cell strains after 24 h of incubation either in 0.03 or 3% FCS/RPMI. RNA was reverse transcribed and relative tenascin-C and c-fos mRNA levels were detected as described [12,13]. Relative mRNA levels for the genes listed in Table 1, normalized to Gapdh, were measured using Platinum® SYBR® Green qPCR SuperMix-UDG with ROX (Invitrogen) and the primers listed in Additional file 4: Table S4. Real-time PCR was performed in a StepOnePlus Real-Time PCR System (Applied Biosystems, Rotkreuz, Switzerland) using a standard cycling profile. All samples were run in duplicate. Data were analyzed by the ∆Ct method [83] and presented as fold changes in mRNA expression levels between HC11-FL and HC11-∆SAP cells. RNA from stretched cells was analyzed by qRT-PCR using the efficiency ∆∆Ct method [84] that included a further normalization to the resting control. Data represent means ± SD from three independent experiments.

### Protein analyses by immunoblotting and zymography

After 24 h of starvation, whole-cell extracts from the three HC11 strains were prepared in RIPA buffer and immunoblotting was performed as described [12,13]. The following primary antibodies were used: mAb65F13 anti-Mkl1 [12], MTn12 anti-Tnc [85], anti-Wisp1/CCN4 (clone 214203, R&D Systems), anti-Nox4 (NB110-58851, Novus Biologicals), anti-Vcl (clone hVIN-1, Sigma) and anti-Gapdh (ab9485, Abcam).

After reaching 90% confluency, HC11 strains were starved for 48 h before conditioned medium was collected, concentrated and analyzed by zymography as described [86].

### Promoter-reporter assays

The tenascin-C promoter used in this study was described as TNC 247 bp [13]. Promoters of Acta2 [87] and all SRF-independent/SAP-dependent genes described in Table 1 were PCR-amplified from genomic DNA and corresponded to the sequences listed in Additional file 4: Table S5. Each promoter contained ≥ 500 bp 5′ of the TSS and was cloned into the pSEAP2-Basic (Clontech, Saint-Germain-en-Laye, France). For some promoters also 200 bp proximal promoter sequences were cloned as described above. All clones were verified by DNA sequencing.

HC11 cells in 6-well plates were cotransfected with 1 μg of the SEAP reporter vectors, 1 μg of pcDNA3 vectors encoding Mkl1 variants [13], and 200 ng of the secreted luciferase MetLuc vector (Clontech) used to normalize for transfection efficiency. Cells were cultured in 0.03% FCS/RPMI for 24 h before enzymatic activity measurements were performed as described [13]. Experimental values represent averages of three independent experiments, each performed in duplicate.

### Statistical analysis

Numerical results were expressed as means ± SD. Statistical analysis was completed using GraphPad InStat Software, version 3.05. The two-tailed Student’s *t* test was used to evaluate differences between two groups. Multiple comparisons were performed using one-way analysis of variance (ANOVA). Values of P less than 0.05 were considered statistically significant. Statistics for bioinformatics analyses is given in figure legends.

## Competing interests

The authors declare that they have no competing interests.

## Authors’ contributions

MBA and RCE conceived the project. MBA designed the experiments. MBA and TR performed transcript profiling of HC11 cell strains and bioinformatics analysis. Promoter-reporter studies were designed by RCE and performed by JF. IG and JF performed Western blot and zymographic analysis, and mechanical strain experiments. IG performed qRT-PCR experiments, cell proliferation and cell migration assays. MBA and RCE interpreted the data and wrote the paper. All authors discussed the results, read and approved the final manuscript.

## Supplementary Material

Additional file 1: Table S1 SRF-dependent/SAP-independent probeset list. Click here for file

Additional file 2: Table S2 SRF-dependent/SAP-dependent probeset list. Click here for file

Additional file 3: Table S3 SRF-independent/SAP-dependent probeset list. Click here for file

Additional file 4: Table S4 Primer sequences. **Table S5.** Promoter constructs. **Figure S1.** Quantification of SAP-dependent Mkl1 target gene expression using qRT-PCR analysis. **Figure S2.** Differential expression of newly discovered Mkl1 target genes in HC11 strains overexpressing either FL-, mutB1- or ΔSAP-Mkl1 constructs (protein analyses performed by immunoblotting and zymography). **Figure S3.** SAP-dependent Mkl1 target genes are correlated with a very high significance (P < 0.00001) with the two proliferation modules – mitotic checkpoint and mitotic progression (a functional correlation analysis performed using the GOBO bioinformatics tool). Click here for file
