# Supplementary material for: SAP domain-dependent Mkl1 signaling stimulates proliferation and cell migration by induction of a distinct gene set indicative of poor prognosis in breast cancer patients
Source: Mol Cancer. 2014 Feb 5;13:22. doi: 10.1186/1476-4598-13-22 (PMC3933235; doi:10.1186/1476-4598-13-22)
Supplement: Additional file 4: Table S4 — Primer sequences. Table S5. Promoter constructs. Figure S1. Quantification of SAP-dependent Mkl1 target gene expression using qRT-PCR analysis. Figure S2. Differential expression of newly discovered Mkl1 target genes in HC11 strains overexpressing either FL-, mutB1- or ΔSAP-Mkl1 constructs (protein analyses performed by immunoblotting and zymography). Figure S3. SAP-dependent Mkl1 target genes are correlated with a very high significance (P < 0.00001) with the two proliferation modules – mitotic checkpoint and mitotic progression (a functional correlation analysis performed using the GOBO bioinformatics tool). [file 1476-4598-13-22-S4.docx]

**Additional file 4 for:**

**SAP domain-dependent Mkl1 signaling stimulates proliferation and cell migration by induction of a distinct gene set indicative of poor prognosis in breast cancer patients**

Irem Gurbuz^1, 2^, Jacqueline Ferralli^1^, Tim Roloff^1^, Ruth Chiquet-Ehrismann^1, 2^ and Maria B. Asparuhova^1^

^1^Friedrich Miescher Institute for Biomedical Research, Basel, Switzerland and ^2^University of Basel, Basel, Switzerland

**Content:**

**Table S4.** Primer sequences.

**Table S5.** Promoter constructs.

**Figure S1.** Quantification of SAP-dependent Mkl1 target gene expression using qRT-PCR analysis.

**Figure S2.** Differential expression of newly discovered Mkl1 target genes in HC11 strains overexpressing either FL-, mutB1- or ΔSAP-Mkl1 constructs (protein analyses performed by immunoblotting and zymography).

**Figure S3.** SAP-dependent Mkl1 target genes are correlated with a very high significance (P < 0.00001) with the two proliferation modules – mitotic checkpoint and mitotic progression (a functional correlation analysis performed using the GOBO bioinformatics tool).

**Table S4. Primer Sequences.**

| **Gene symbol** | **Gene bank accession**  **number** | **Primer pair (fwd/rev)** | **Amplicon**  **size (bp)** |
| --- | --- | --- | --- |
| Anln | NM_028390.3 | 5’-cagtggtgacgctctgacat-3'  5’-gggactggccataactgaag-3' | 220 |
| Nox4 | NM_015760.4 | 5’-cattccagtggtttgcagatt-3'  5’-aactgggtccacagcagaaa-3' | 249 |
| Adamts16 | NM_172053.2 | 5’-ttggagagaaagccaagctc-3'  5’-gggccttcatcaccgtact-3' | 181 |
| Krt5 | NM_027011.2 | 5’-caggacctggtggaggacta-3'  5’-ccatggaaaggaccacagat-3' | 241 |
| p15(PAF) | NM_026515.2 | 5’-gggaattcttcaggctgtcc-3'  5’-caacaagccaattggacaaa-3' | 227 |
| Ass1 | NM_007494.3 | 5’-caccacatccctggaactct-3'  5’-atgagcgtggtaaaggatgg-3' | 151 |
| Cd34 | NM_001111059.1 | 5’-aggctgatgctggtgctagt-3'  5’-actccagaggtgaccaatgc-3' | 235 |
| Wisp1 | NM_018865.2 | 5’-gctctaccacctgtggccta-3'  5’-acagcctgcgagagtgaagt-3' | 194 |
| Mcm6 | NM_008567.1 | 5’-catgtcccgctttgatctct-3'  5’-ctggcggagacgtttgtact-3' | 232 |
| Car12 | NM_178396.4 | 5’-gttcgatgagaggctggtgt-3'  5’-cctcagcctccttcttgatg-3' | 215 |
| Htatip2 | NM_016865.3 | 5’-gctggatgtctatgcttctgc-3'  5’-tcaaccttggcttccacttc-3' | 243 |
| Kif26b | NM_001161665.1 | 5’-aagaagcagccaggttcctc-3'  5’-aatgcccaggttctgcatag-3' | 214 |
| Lox | NM_010728.2 | 5’-cagggattgagtcctggatg-3'  5’-actgggaactgggcttcttt-3' | 242 |
| Mmp12 | NM_008605.3 | 5’-catcccatctggtattcaagc-3'  5’-atgagctcctgcctcacatc-3' | 249 |
| Mmp3 | NM_010809.1 | 5’-catcaccaatgtgcagctct-3'  5’-ctcctcgtgccctcgtatag-3' | 248 |
| Gapdh | NM_008084.2 | 5’-CTTGTGCAGTGCCAGCCTC-3'  5’-GCCGTGAGTGGAGTCATACTG-3' | 189 |

**Table S5. Promoter constructs.**

| **Promoter** | **Accession number** | **Nucleotide position** |
| --- | --- | --- |
| mTNC 247bp | NT_039260.7 | 3336826-3336433 |
| Wisp1 >500bp | NT_039621.7 | 28008443-28009129 |
| Wisp1 200bp | NT_039621.7 | 28008801-28009129 |
| Krt5 >500bp | NT_039621.7 | 62829844-62829249 |
| Krt5 200bp | NT_039621.7 | 62829551-62829249 |
| Kif26b >500bp | NT_039185.7 | 22360019-22360523 |
| Kif26b 200bp | NT_039185.7 | 22360158-22360523 |
| Htatip2 >500bp | NT_039424.7 | 10657737-10658750 |
| Htatip2 200bp | NT_039424.7 | 10658493-10658750 |
| Nox4 >500bp | NT_039433.7 | 4994940-4995484 |
| Nox4 200bp | NT_039433.7 | 4995123-4995484 |
| Car12 >500bp | NT_039474.7 | 12981551-12982239 |
| Car12 200bp | NT_039474.7 | 12981860-12982239 |
| Adamts16 >500bp | NT_039589.7 | 16522290-16521672 |
| Adamts16 200bp | NT_039589.7 | 16522032-16521672 |
| Cd34 >500bp | NT_039190.7 | 2888907-2889501 |
| Cd34 343bp | NT_039190.7 | 2889159-2889501 |
| Anln >500bp | NT_039472.7 | 8976459-8975867 |
| p15(PAF) >500bp | NT_039474.7 | 12158197-12158785 |
| Mcm6 >500bp | NT_078297.6 | 42913044-42912468 |
| Ass1 >500bp | NT_039206.7 | 8927941-8928521 |
| Acta2 562bp | NT_039687.7 | 27442283-27441676 |


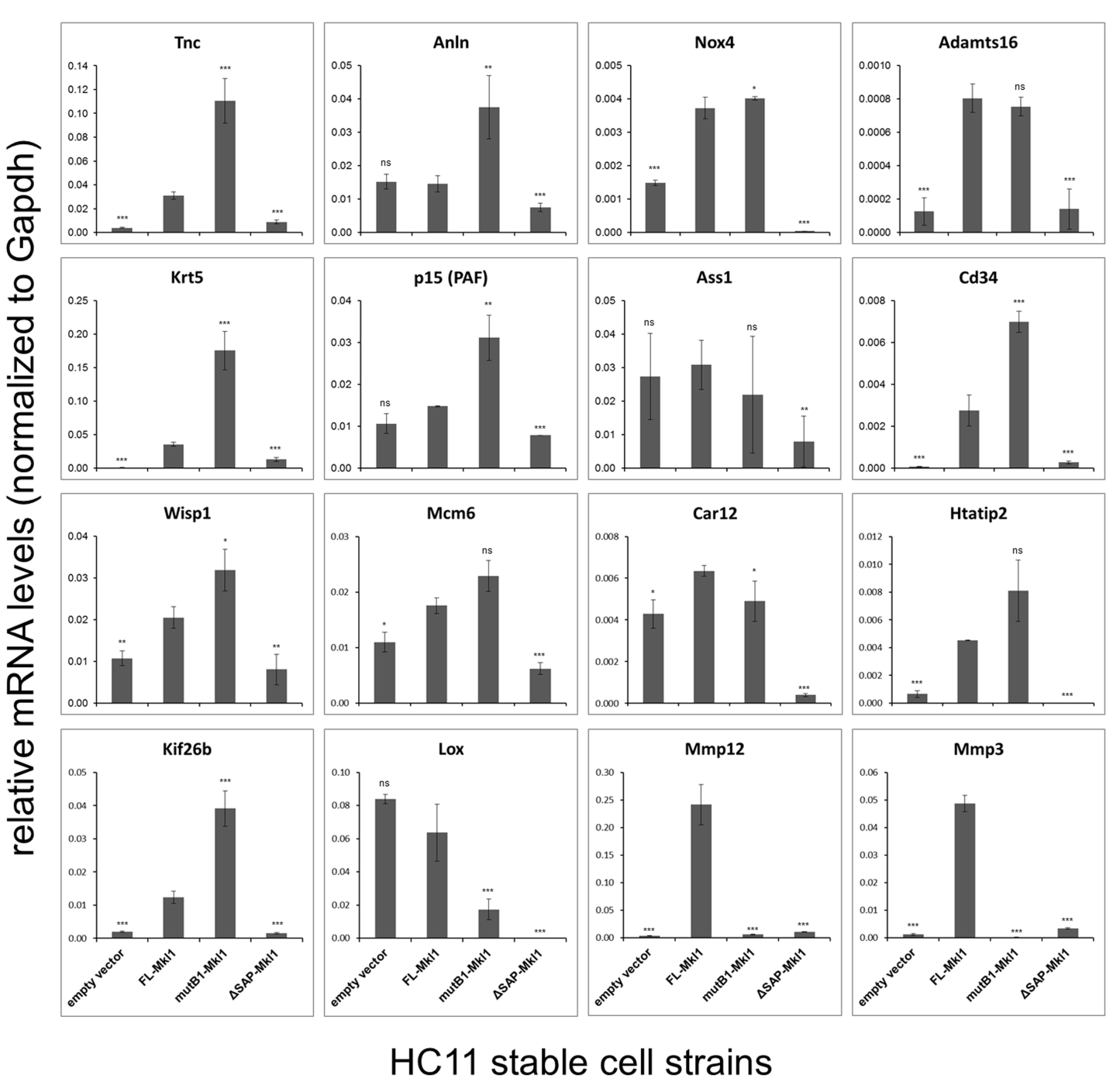


**Figure S1.** **Quantification of SAP-dependent Mkl1 target gene expression using qRT-PCR analysis.** Relative mRNA levels for the genes listed in Table 1, normalized to Gapdh, were analyzed in HC11 cells stably transfected with either empty vector or with vectors encoding FL-, mutB1- or ΔSAP-Mkl1 proteins. The results agreed with the data obtained by transcript profiling (Figure 1). Induction of these genes strongly depends on the SAP domain of Mkl1 as well as on the B1 domain for Lox, Mmp12 and Mmp3 genes, which belong to the SRF-dependent/SAP-dependent gene set. Means ± SD from three independent experiments and significant differences to the HC11-FL cells, ***P < 0.001, **P < 0.01, *P < 0.05 are shown.

**
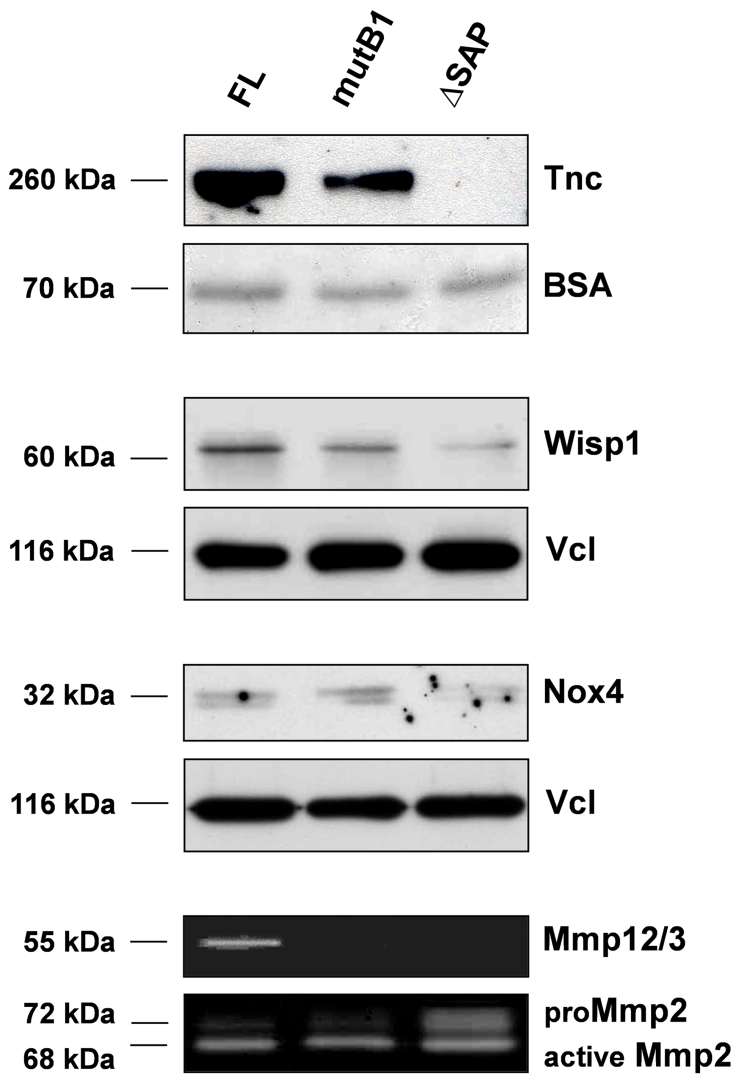
**

**Figure S2. Differential expression of newly discovered Mkl1 target genes in HC11 strains overexpressing either FL-, mutB1- or ΔSAP-Mkl1 constructs.** Western blot analysis of Tnc, Wisp1 and Nox4, and zymographic analysis for Mmp12/3 show SAP-dependent protein expression in HC11 epithelial cells. Secreted Tnc protein was detected in cell culture media using the MTn12 anti-Tnc antibody, and bovine serum albumin (BSA) from the medium visualized by Ponceau S staining served as loading control. Wisp1 and Nox4 proteins were detected in whole-cell extracts from the three HC11 cell strains, HC11-FL, HC11-mutB1 and HC11-ΔSAP using respective antibodies. Anti-Vcl served as loading control. Conditioned media from the three HC11 cell strains was subjected to casein or gelatin zymography for detection of the matrix metalloproteinases Mmp12/3 and Mmp2, respectively. For Mmp2, both an inactive (proMmp2) and active form was detected and served as loading control. Relevant bands and molecular weight markers (in kDa) are indicated on the right and left of each panel, respectively.

**
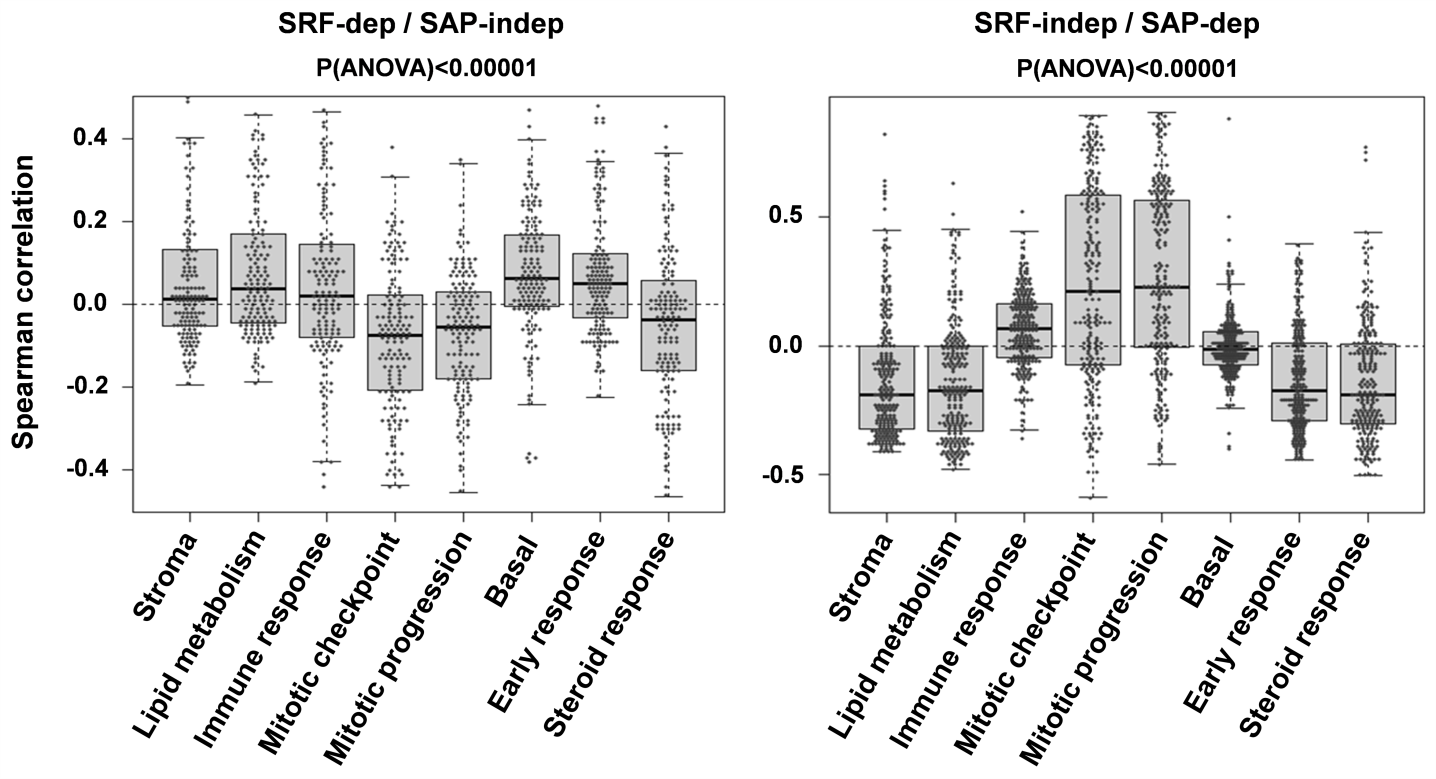
**

**Figure S3. SAP-dependent Mkl1 target genes are correlated with a very high significance (P < 0.00001) with the two proliferation modules – mitotic checkpoint and mitotic progression.** Functional correlation of genes in the SRF-dependent/SAP-independent (left panel) and SRF-independent/SAP-dependent (right panel) gene sets to different gene expression modules emulating breast cancer-specific biological processes [33] was performed using the GOBO bioinformatics tool. For each gene module and gene in the two gene data sets, a Spearman correlation value is computed by comparing the expression pattern across all samples for a specific gene to the corresponding rank sum for each sample in the specific module. Dots indicate actual correlation values.
